# (Mis)placed central venous catheter in the left superior intercostal vein

**DOI:** 10.2478/v10019-010-0043-7

**Published:** 2010-09-29

**Authors:** Ranka Stern Padovan, Maja Hrabak Paar, Igor Aurer

**Affiliations:** 1 Department of Diagnostic and Interventional Radiology, University Hospital Center Zagreb, University of Zagreb, School of Medicine, Zagreb, Croatia; 2 Department of Internal Medicine, Division of Hematology, University Hospital Center Zagreb, University of Zagreb, School of Medicine, Zagreb, Croatia

**Keywords:** central venous catheterization, helical computed tomography, lymphoma, non-Hodgkin

## Abstract

**Background:**

Chest X-ray is routinely performed to check the position of the central venous catheter (CVC) inserted through the internal jugular or subclavian vein, while the further evaluation of CVC malfunction is usually performed by contrast venography. In patients with superior vena cava obstruction, the tip of the catheter is often seen in collateral mediastinal venous pathways, rather than in the superior vena cava. In such cases detailed knowledge of thoracic vessel anatomy is necessary to identify the exact location of the catheter.

**Case report.:**

We report a case of 32-year-old female patient with relapsing mediastinal lymphoma and previous superior vena cava obstruction with collateral azygos-hemiazygos venous pathways. The patient had CVC inserted through the left subclavian vein and its position was detected by CT to be in the dilated left superior intercostal vein and accessory hemiazygos vein. Considering that dilated accessory hemiazygos vein can tolerate infusion, the CVC was left in place and the patient had no complaints related to CVC (mal)position. Furthermore, we present anatomical and radiological observations on the azygos-hemiazygos venous system with the special emphasis on the left superior intercostal vein.

**Conclusions:**

Non-contrast CT scans can be a valuable imaging tool in the detection of the CVC position, especially in patients with renal insufficiency and contrast media hypersensitivity.

## Introduction

Chest X-ray is routinely performed to ascertain the position of the central venous catheter (CVC). The catheter should be placed in a large-calibre vein with a sufficient flow to tolerate infusion which is usually the subclavian vein, brachiocephalic vein or superior vena cava.^1^ If further investigation of CVC position or malfunction is needed, contrast venography is usually performed, with intravenous contrast media administration under the fluoroscopic control using the digital subtraction angiography technique nowadays.^2^ It is performed also in different vein pathologies.^3^

Radiographic and venographic features of CVC malposition in the left superior intercostal vein were previously described.^4^–^6^ We report a case of a patient in whom CVC position was correctly detected to be in the left superior intercostal vein using non-contrast computed tomography (CT). Thus, the correct interpretation of CVC position obviated contrast venography.

## Case report

**FIGURE 1.**
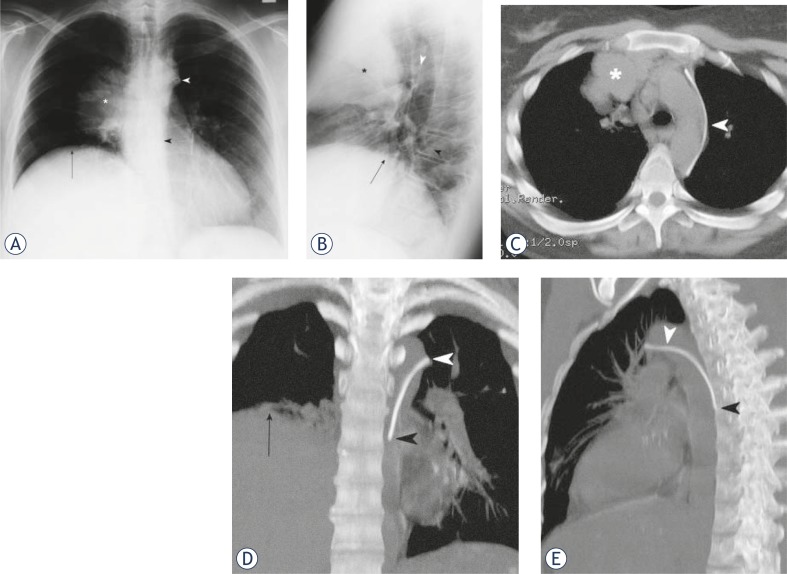
Posteroanterior (A) and lateral (B) chest X-ray. Axial (C), coronal (D) and sagittal (E) maximum intensity projection (MIP) of non-enhanced thoracic CT scan. Catheter inserted through the left subclavian vein arcs laterally in the left superior intercostal vein (white arrowhead) and then descends medially in the posterior mediastinum through the accessory hemiazygos vein (black arrowhead). Bulky mediastinal mass (asterisk) causing right phrenic nerve palsy and elevation of the right hemidiaphragm (arrow).

A 32-year-old woman presented to our hospital with relapsing bulky mediastinal non-Hodgkin lymphoma and previous superior vena cava obstruction. A single-lumen CVC was uneventfully inserted through the left subclavian vein without imaging guidance. Aspirated blood was venous. Routine chest X-ray was obtained to ascertain the position of the catheter (Figures 1A, B) and was interpreted as showing the catheter in the descending aorta. The right upper mediastinal mass with consequent right phrenic nerve palsy and elevation of the right hemidiaphragm was also evident.

Since hemodynamic parameters were consistent with a venous localization of the catheter, it was left in place and three hours later a non-contrast thoracic CT scan (GE LightSpeed Ultra, General Electric Healthcare, Milwaukee, USA) was performed in order to clarify its position (Figures 1C, D, E). It showed the CVC passing through the left subclavian vein, arching laterally along the aortic arch through the left superior intercostal vein and then descending medially in the posterior mediastinum through the accessory hemiazygos vein. Superior vena cava and both brachiocephalic veins were narrowed and incorporated into the bulky mediastinal mass with developed collateral pathways via the azygos-hemiazygos system. Considering that the dilated accessory hemiazygos vein can tolerate infusion, the CVC was left in place and functioned well for three weeks. During that period the patient had no chest or back pain, nor other complaints related to CVC (mal)position.

## Discussion

Malposition occurs in about one-third of CVC insertions if imaging guidance is not used, while it happens less frequently under sonographic or fluoroscopic guidance.^1^ One of the rare courses of the misplaced catheter is the left superior intercostal vein, accessory hemiazygos vein and hemiazygos vein.

The left superior intercostal vein and azygos arch present a major venous loop connecting the posteriorly located azygos-hemiazygos system with the anteriorly located superior vena cava and left brachiocephalic vein.^7^ They develop from the same embryological structures – the main tract from the supracardinal vein and the terminal segment from the posterior cardinal vein.^8^ The left superior intercostal vein collects blood from the left second through fourth intercostal vein, and at the level of T3 or T4 it courses anteriorly along the lateral wall of the aortic arch and empties into the left brachiocephalic vein near the venous angle. In 75% of individuals it communicates with the accessory hemiazygos vein that drains fifth through eighth left posterior intercostal veins. The accessory hemiazygos vein may communicate with the hemiazygos vein at the level T8 or T9 where hemiazygos vein crosses the midline to join the azygos vein.^9^ If the left superior vena cava persists, the left superior intercostal vein becomes its tributary, analogous to the azygos arch on the right.^1^

The left superior intercostal vein can be identified on upright posteroanterior chest radiograph in 1.4%–9.5% of healthy people as a small “nipple” lateral to the aortic arch.^9^,^10^ Diameter of the “aortic nipple” in healthy patients measures up to 4.5 mm on erect chest radiograph, and is 1–2 mm larger in supine position.^10^ In case its diameter exceeds 4.5 mm, the patient should be investigated for a possible underlying venous abnormality. Dilatation of the left superior intercostal vein can be congenital or acquired. It is usually caused by superior or inferior vena caval obstruction/anomalies, congestive heart failure, portal hypertension, Budd-Chiari syndrome and left brachiocephalic vein hypoplasia.^10^ In case of superior vena cava syndrome, its detection on chest X-ray can precede clinical symptoms by seven to ten weeks.^11^ The course of the catheter in the left superior intercostal vein can be distinguished on chest X-ray from that of the catheter in a persistent left superior vena cava or in a pericardiophrenic vein because the latter two descend along left side of the mediastinum without arcing laterally along the aortic arch.^1^

The non-dilated left superior intercostal vein is usually too narrow for the insertion of a CVC. The insertion of the CVC tip into the narrow left superior intercostal vein irritates the vessel wall leading to catheter-related infection and thrombosis. Our patient had prior superior vena cava syndrome and thrombosis, and consequently developed collateral venous pathways, including the enlargement of the left superior intercostal vein. Enlarged collateral veins can occasionally be the site of CVC malposition. The CVC was left in place in the left superior intercostal and accessory hemiazygos vein because developed collateral venous pathways usually have a sufficient flow to tolerate infusion. The deliberate placement of CVC into the azygos-hemiazygos system with the effective long-term venous access in the patient with superior vena caval occlusion was previously reported.^12^

When oncologic patients undergo follow-up CT examinations of their underlying disease process^13^, it is important to be aware of the CT findings that indicate malpositioning of the catheter.^14^ Detailed knowledge of normal and anomalous venous anatomy is necessary for the correct interpretation of the CVC position. Because of the high radiation dose, CT scan cannot be recommended as a routine diagnostic procedure for the CVC position detection, but we believe that if there is any other indication for thoracic CT scanning, e.g. evaluation of mediastinal lymphadenopathy or lung disease, the position of the catheter can be precisely determined based on CT exam, making contrast venography unnecessary. The main disadvantage of the CT scan versus contrast venography is its inability to reposition the misplaced catheter. In addition, non-contrast CT scanning can be of use in patients with renal failure and contrast media hypersensitivity. Such scans are usually sufficient for the detection of CVC tip position in mediastinal veins, which are not accessible to colour Doppler imaging because of the restricted acoustic window.

## Conclusions

CT can be a valuable tool in evaluation of the CVC position, especially in patients in whom diagnostic contrast venography is contraindicated due to renal failure or contrast media hypersensitivity. If the CVC tip is detected to be in the dilated left superior intercostal vein or accessory hemiazygos vein in patients with superior vena caval syndrome, the vein can usually tolerate infusion and the catheter can be left in place. Detailed knowledge of normal and anomalous venous anatomy is required for the optimal interpretation of the CVC position.
